# Prevalence of Chronic Bronchitis and Respiratory Health Profile of a Population Exposed to Wood Smoke in Nicaragua

**DOI:** 10.5696/2156-9614-10.26.200607

**Published:** 2020-05-26

**Authors:** Annika Maas, Henning Kothe, Ivette Pilarte Centeno, Mauricio José Gutiérrez Leiva, Klaus Dalhoff

**Affiliations:** 1 University of Luebeck, Luebeck, Germany; 2 Faculty of Medical Sciences, National Autonomous University of Nicaragua (UNAN), Managua, Nicaragua

**Keywords:** HAP, household air pollution, respiratory health, wood smoke exposure, Nicaragua, lung function, chronic bronchitis, COPD

## Abstract

**Background.:**

Household air pollution (HAP) is one of the most important environmental risk factors worldwide associated with chronic respiratory diseases.

**Objectives.:**

The present study focused on respiratory health in a population with high wood smoke exposure in Nicaragua.

**Methods.:**

We employed a cross-sectional study with 213 participants. Data on the prevalence of chronic bronchitis (chronic bronchitis), chronic obstructive pulmonary disease (COPD) and asthma, including respiratory scores and pulmonary function tests, were documented. The role of risk factors for chronic bronchitis was analyzed.

**Results.:**

We found a high prevalence of chronic airway diseases in the population exposed to wood smoke. A higher prevalence of chronic bronchitis was found in persons serving as primary cooks in households. Further confounding factors for chronic bronchitis included age, a prior diagnosis of asthma, inhalational allergies and lower socioeconomic status. Respiratory scores were elevated in individuals with chronic bronchitis.

**Conclusions.:**

This is one of the first studies in a wood smoke-exposed population in Nicaragua showing a high prevalence of chronic bronchitis and COPD with an emphasis on the analysis of personal and environmental risk factors. Further studies are needed to address which combination of interventions is most efficient for ameliorating respiratory health hazards.

**Participant Consent.:**

Obtained

**Ethics Approval.:**

The study protocol was approved by the Ethics Committee of the University of Luebeck, Germany (reference number 12-214), and by the Ethics Committee of the Department of Medical Sciences at National Autonomous University of Nicaragua, Managua, Nicaragua.

**Competing Interests.:**

The authors declare no competing financial interests.

## Introduction

Worldwide, 2.7 billion people (38% of the world population) use solid biomass fuels for daily food preparation.^1^ Cooking with biomass fuels on inefficient fireplaces or cook stoves for a prolonged time is an important cause of household air pollution (HAP), especially when the cooking is done in poorly ventilated areas.^2^,^3^ Previous studies have shown high levels of particulate matter and carbon monoxide during food preparation under such conditions.^4^ Household air pollution is known to be one of the most important environmental risk factors worldwide and has been shown to be responsible for more than 3 million deaths in 2012.^5^,^6^ Globally it is an important cause of chronic obstructive lung disease (COPD) and is associated with further health issues such as arterial hypertension and pneumonia in children.^5^,^7^

The present study focused on the association between HAP and respiratory diseases in the Latin American country of Nicaragua, where 52.7% of the households uses wood as fuel source for food use.

Nicaragua is one of the three poorest countries in Latin America.^8^ The population is young, with a mean age of 24.7 years and a life expectancy of 76.9 years for men and 80.7 years for women.^9^,^10^ The fertility rate is 2.5 children per woman (15–49 years) and the rate of premature birth is 6.7%, which is of interest because of the association between low birth weight and reduced lung function in children and adults.^11^,^12^ The infant mortality rate in the first year of life is 17/1000 and the biggest health issues for children under five years are gastroenteritis, acute respiratory infections and parasitic diseases (year 2013).^11^ According to the World Health Organization (WHO), the most common causes for premature death for adults in Nicaragua, expressed as years of life lost, are lower respiratory infections, ischemic heart diseases, congenital anomalies and complications from preterm birth.^13^

Due to the tropical climate there is no need for heating in Nicaragua, which is in contrast to non-tropical Asian or African regions where wood smoke exposure includes heating with firewood in addition to cooking.^14^ A Nicaraguan cross-sectional study observed high concentrations of carbon monoxide and particulate matter (PM)_2.5_ among individuals exposed to open fire, which are presumed to be responsible for inflammatory processes in the lung.^4^,^15^,^16^ There are few studies on respiratory health in Nicaragua.^4^ Data on the prevalence of COPD and asthma in the adult population are available from the Global Burden of Disease (GBD) study 2017.^17^

There are few data on the influence of wood smoke exposure on respiratory health and chronic bronchitis in Latin American countries. The randomized controlled Randomized Exposure Study of Pollution Indoors and Respiratory Effects (RESPIRE) study in Guatemala found a consistent reduction in risk for respiratory symptoms in a stove intervention group compared to persistent open wood fire use after a follow-up of 18 months.^18^ A cross-sectional study in Colombia found a higher prevalence of chronic bronchitis in wood smoke exposed individuals compared to non-exposed controls with an OR of 1.44 (95% CI 1.09–1.90) and 53.6% of chronic bronchitis cases in women occurred in non-smokers.^19^ A cross-sectional study in Guatemala with wood smoke exposed women showed a correlation between respiratory symptoms and the level of exposure as measured by carbon monoxide in exhaled breath, but not with reduced lung function.^20^ The present study evaluated the prevalence of chronic bronchitis, COPD and asthma, including respiratory scores and lung function tests in a cohort of participants living in regions with high exposure to wood fire smoke in Nicaragua.

**Abbreviations**
CAT: Chronic obstructive pulmonary disease Assessment Test
COPD: Chronic obstructive pulmonary disease
FEV1: Forced expiratory volume in one second
FVC: Forced vital capacity
HAP: Household air pollution
mMrc: Modified Medical Research Council
PM: Particulate matter
WHO: World Health Organization

## Methods

**Figure 1:**
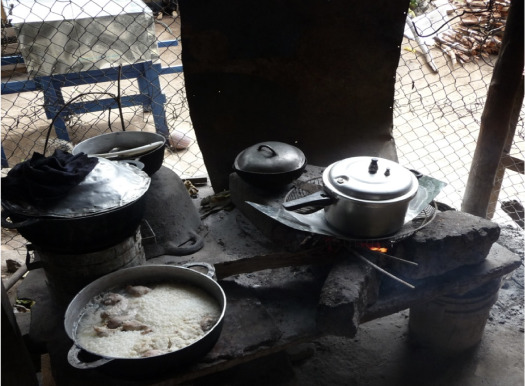
Open fireplace at the study site of Granada

This was a cross-sectional study conducted in two municipalities and one township in the states of Granada and Managua in central Nicaragua. Altitude in the study area ranges between 60 m and 340 m above sea level. A total of 80 households participated in the study. There was cooperation with the local NGO, Fuprosomunic, and the medical faculty of the University of Managua who had performed a socio-epidemiological study in one of the study locations in 2011.^21^ Participants in the study areas live in stone or adobe houses with corrugated metal roofs. Kitchens are detached from living areas and are constructed as a semi-open space with a roof and partial walls (*Figure 1*). Cooking fires are generally a stone construction, surrounded by either three stones or an adobe construction to place the pot on (*Figure 1*). There are no proper ventilation devices such as chimneys.

We defined two domicile groups in order to compare local living conditions, as socioeconomic status was higher and total wood use lower in Ticuantepe (designated Region I) versus Granada/Diriomo (designated Region II).

The percentage of the population using wood as a fuel source was 24.7% for Region I and 54.6% for Region II.^11^,^2^–^24^ The rate of severe poverty was higher in Region II (30.5%) compared to Region I (16.8%).^22^–^24^

Eligibility criteria included age > 16 years and regular use of wood fires for cooking in the household. Exclusion criteria were the use of fuel sources other than wood, pulmonary diseases not related to inhalational hazards, acute respiratory infections and clinical signs of neoplasia or systemic inflammatory disorders (i.e. unexplained weight loss, night sweats, fever). All participants gave oral and written informed consent by signature or fingerprint. The study protocol was reviewed and approved by the Ethics Committee of the University of Luebeck, Germany (reference number 12–214), and by the Ethics Committee of the Department of Medical Sciences at National Autonomous University of Nicaragua, Managua, Nicaragua.

### Assessments

Questionnaire-based interviews were performed using a modified version of the respiratory questionnaire described in the RESPIRE study (Supplemental Material).^18^ We added the COPD Assessment Test (CAT), Modified Medical Research Council (mMRC) dyspnea scale and additional questions on local living conditions and the level of daily wood smoke exposure according to daily cooking procedures. Enhanced exposure was defined as serving as the primary household cook at open wood fireplaces (minimum exposure of one hour daily, mean exposure time of 2.9 hours/day). Spirometry was done at a central study place in each of the three study locations (in two regions), using a laptop-based hand spirometer (MasterScope by Carefusion). According to American Thoracic Society and the European Respiratory Society at least three spirometries need to be performed successfully per individual to fulfil reproducibility criteria.^25^ Data collection took place from February until April 2013.

### Endpoints

The main questions addressed the prevalence of chronic bronchitis as defined by cough with sputum expectoration for at least three months/year during a period of two consecutive years and prevalence of COPD defined by a fixed forced expiratory volume in one second (FEV1)/forced vital capacity (FVC) ratio < 0.7. Bronchodilatation before spirometry was not possible to perform due to logistical reasons. In order to compare our data with the general population in Nicaragua we chose data from the available literature on respiratory health from Nicaragua. Asthma was defined as a prior diagnosis of asthma by physician/medical staff plus clinical symptoms such as wheezing and/or chest tightness.

We furthermore analyzed the role of confounding factors such as age, inhalational allergies, level of wood smoke exposure and local living conditions for the prevalence of chronic bronchitis.

### Statistical analysis

Descriptive statistics were done using SPSS version 19.9. Normality of measured spirometry data was assessed using the Kolmogorov– Smirnov test. Respiratory scores were compared using the Mann Whitney U test. Outcomes of non-parametric data as risk factors for chronic bronchitis were compared using the Chi-square test.

## Results

Only 213/318 of the screened subjects participated in the present study and the main reasons for screening failure were a distant working place and personal denial of participation. Mean age ± SD was 37.7 ± 17.5 years, the male/female ratio was 32.9/67.1%, 12.3% were tobacco smokers, and 24.2% were passive smokers in the study population. The rate of obesity, defined as a body mass index above 30, was 27.5% (n= 57) (data not shown in Tables).

The prevalence of chronic bronchitis in the study population was high without major differences between men and women, but markedly increased with age (*Table 1*). The prevalence of chronic obstructive pulmonary disease as diagnosed according to spirometric criteria was 6.3% for the total study population and for women and also increased with age. Of the individuals with COPD, 7/12 (around 58%) had a FEV1 <= 80.0% and > 50.0%, consistent with moderate severity, the other 5/12 had a FEV1 > 80.0%. The prevalence of asthma was 10.2% in the total study population and 12.2% in women, both increased with age (*Table 1*).

**Table 1:** Prevalence of Chronic Bronchitis, COPD and Asthma

**Prevalence**	**Study population**	

	**Total N= 213 N (%)**	**Women N= 143 N (%)**
Chronic Bronchitis		
All ages	35 (16.7)	23 (16.3)
15–49 years	18 (12.2)	11 (11.6)
50–69 years	14 (25.9)	10 (25.0)
COPD		
All ages	12 (6.3)	8 (6.3)
15–49 years	4(3.0)	2 (2.4)
50–69 years	5 (9.6)	3 (7.9)
Asthma		
All ages	21 (10.2)	17 (12.2)
15–49 years	10 (7.0)	8 (8.6)
50–69 years	9(16.4)	7(17.5)

A total of 197 participants received spirometry, which fulfilled American Thoracic Society and the European Respiratory Society quality criteria (three successful spirometries) in 191 cases.^25^ (*Table 2*).

**Table 2:** Spirometric Data (N=191)

**Value**	**Study population**	

	**Males N= 63**	**Females N= 128**
FVC (L)	4.36 ± 0.7	2.97 ± 0.65
FEV1 (L)	3.51 ± 0.6	2.44 ± 0.56
FEV1/FVC (%)	80.36 ± 6.28	82.21 ± 7.1
Peak expiratory flow rate (L)	7.27 ± 1.38	5.09 ± 1.29

Mean values ± SD.

The respiratory scores used in the study population revealed significantly higher values in participants with chronic bronchitis compared to individuals without chronic bronchitis (*Table 3*). Individuals with chronic bronchitis were more often in the elevated mMRC groups 2-4 compared to individuals without chronic bronchitis.

**Table 3:** Results of the mMRC and CAT Score According to the Presence of Chronic Bronchitis; N= 209/213

**Score**	**Total** N= 209^*^	**No chronic bronchitis** N= 174^*^	**Chronic bronchitis** N= 35^*^	**p-value**
mMRC score	0.73 ± 1.07	0.63 ± 0.96	1.26 ± 1.44	0.01
CAT score	5.85 ± 7.65	4.41 ± 5.65	12.71 ± 11.8	0.00

^*^Mean values ± SD.

Analysis per Mann-Whitney U Test.

p-value: Individuals with chronic bronchitis compared to those without chronic bronchitis.

Table 4 shows the prevalence of chronic bronchitis by selected risk factors and confounding variables. Significant confounders were age (> 50 years), serving as the primary household cook using a wood fire for food preparation, prior diagnosis of asthma, inhalational allergies and residence in Region II. A total of 91.4% cases of chronic bronchitis occurred in non-smokers and 100% of chronic bronchitis cases occurred in non-smokers for females (data not shown). Neither smoking status nor sex or obesity had a confounding influence on the prevalence of chronic bronchitis.

**Table 4:** Prevalence of Chronic Bronchitis by Selected Variables (N= 213)

**Variables**	**Categories**	**N**	**%**	**p-value**
Sex	Male	12	17.6	NS
	Female	23	16.3	
Age	<50	18	12.2	0.01
	>= 50	17	27.4	
Body mass index >30	No	26	17.6	NS
	Yes	8	14.5	
Smoking status	Non-smoker	32	17.4	NS^*^
	Smoker	3	12.5	
Primary cook using wood fire	No	2	5.4	0.04
	Yes	32	19.5	
Diagnosis of asthma	No	24	13.6	0.003^*^
	Yes	10	40.0	
Inhalational allergies	No	27	14.6	0.04^*^
	Yes	8	33.3	
Residence	Region I	5	5.5	0.00
	Region II	30	25.4	

Analysis with Chi^2^ test.

*Fisher's exact test.

Region I= Ticuantepe, Region II= Granada and Diriomo.

Inhalational allergies= allergies to dust, flour. Missing values, 4.

Abbreviation: NS, not significant.

## Discussion

The data in the present study demonstrate a high prevalence of chronic bronchitis (16.7%) in a Nicaraguan population with high exposure to wood smoke used for food preparation. The prevalence of chronic bronchitis around the world ranges from 3.4% to 22%, with regional differences.^25^–^27^ There is no representative data for Nicaragua on the prevalence of chronic bronchitis. A previous cross-sectional study in Nicaragua evaluating the health of non-smoking women exposed to wood fire smoke during daily cooking reported a prevalence of physician-diagnosed chronic bronchitis of 9.3%, with no reported data on the prevalence of chronic bronchitis by symptoms.^4^ A markedly higher prevalence of chronic bronchitis was observed in the present study compared with previously published literature, which may be because the present study measured self-reported symptoms vs physician diagnosis (*Table 5*).

**Table 5:** Comparison of Prevalence of Chronic Bronchitis, COPD and Asthma in the Study Population and in the Literature

**Prevalence**	**Study population**		**Nicaragua (literature)**		

	**Total N= 213 N (%)**	**Women N= 143 N (%)**	**Total**	**Women**	**Reference**
Chronic bronchitis					
All ages	35 (16.7)	23 (16.3)	-	11 (9.3)^*^	Clark^4^
COPD					
All ages	12 (6.3)	8 (6.3)	2.45%	2.56%	WHO^28^
15–49 years	4 (3.0)	2 (2.4)	1.19%	1.26%	WHO^28^
50–69 years	5 (9.6)	3 (7.9)	7.61%	7.24%	WHO^28^
Asthma					
All ages	21 (10.2)	17 (12.2)	-	15 (12.7)^*^	Clark^4^
All ages			4.23%	4.25%	WHO^28^
15–49 years	10 (7.0)	8 (8.6)	3.09%	3.46%	WHO^28^
50–69 years	9 (16.4)	7(17.5)	2.92%	3.25%	WHO^28^

* Physician-diagnosed.

World Health Organization (WHO)^28^: general population; Clark^4^: women exposed to wood smoke.

Many studies have found that underdiagnosis in chronic bronchitis is generally high; in Colombia an underdiagnosis rate of 50.3% was reported in a large study with 5539 participants comparing physician-diagnosed and symptom-reported diagnoses of chronic bronchitis with no difference by sex, age or smoking status.^19^,^29^ The authors found a lower prevalence of 5.5% for chronic bronchitis in Colombia. The study design included an urban population in five big cities with generally lower wood use, and higher smoking prevalence (18.3% in the Colombia study vs. 12.3% in the total population of the present study), however inclusion criteria (individuals in Colombia over the age of 40) make it difficult to compare data.^19^

One main finding of this study was that of all chronic bronchitis cases, 91.4% were non-smokers. This rate rose to 100% of chronic bronchitis cases for females. These results are in line with other exposure studies showing higher proportions of chronic bronchitis in non-smoking females.^19^,^30^

Individuals with chronic bronchitis had significantly higher mMRC and CAT scores, corroborating the clinical diagnosis and the individual burden of disease. To our knowledge, there are no biomass exposure studies in the literature including respiratory scores in an assessment of clinical symptoms for chronic bronchitis and other airway diseases. A study comparing two respiratory scores among workers in Nicaragua to detect chronic bronchitis found a sensitivity of 79% for the mMRC score and a positive predictive value of 57%.^31^ The mean mMRC value of subjects in the present study with chronic bronchitis of 1.26 (SD 1.44) is comparable to mean values of COPD individuals in the Prevalence Study and Regular Practice, Diagnosis and Treatment, Among General Practitioners in Populations at Risk of COPD in Latin America (PUMA) study of 1.4 (SD 1.3).^32^

The prevalence of COPD in the present study was 6.3% for both the total study population and females, which is higher than the prevalence data from WHO for the general population in Nicaragua of 2.45% and 2.56% for females. For age-adjusted prevalence, we found a similar prevalence compared to data from the WHO, especially for females (7.9% versus 7.24% (WHO)), see Table 5. Because of the limited number of cases (n=3 females with COPD), this result has limitations. The Prepocol study analyzed 5539 individuals >40 years in five Colombian cities and reported a prevalence of 8.9%.^33^ This rate is similar to that in the respective age group (>50 years) from the present study. More than half of the subjects with COPD in the present study had a FEV1 <80%, representing at least moderate lung function decline, which underlines respiratory morbidity in a relatively young population.

No relevant reduction of lung function in the study participants was found, apart from the 12 COPD cases. The data of the present study were in a similar range as data from a healthy male working population and a wood smoke-exposed female population in Nicaragua which were used for comparison since no national reference data were available (*Table 6*). Compared to reference values in Bogota, Colombia in a healthy non-smoking population between 18 and 65 years, values for FVC, FEV1 and FEV1/FVC were lower in individuals in the present study.^35^ Values for male participants were lower than mean values in healthy Colombian men, but comparable to mean values of healthy male Nicaraguan workers.^34^ Clark *et al*. found comparable FEV1 values to those in the present study for wood smoke-exposed Nicaraguan women (2.49 L and 2.44 L) (*Table 6*).^4^

**Table 6:** Comparison of Spirometric Data in the Study Population (N=191) and the Literature

**Value**	**Study population**		**Nicaragua (literature)**	

	**Males N= 63**	**Females N= 128**	**Healthy males N= 214 Quintero**^34^	**Females N= 101 Clark**^4^
FVC (L)	4.36 ± 0.7	not shown	4.30 ± 0.54	n.d.
FEV1 (L)	3.51 ±0.6	2.44 ± 0.56	3.64 ± 0.49	2.49 ± 0.52
FEV1/FVC (%)	80.36 ± 6.28	not shown	84.71 ± 6.30	n.d.

Mean values ± SD.

Abbreviation: n.d., not detected.

Interestingly, half of the individuals with COPD in the present study had a prior diagnosis of asthma. Asthma-COPD overlap syndrome has a wide prevalence range according to different definitions. The PUMA study analyzed asthma-COPD overlap syndrome in four countries in Latin America and found a prevalence of 17.9% in the obstructive population and 26.5% in the COPD population (using the definition of Tiffeneau <70% + medical diagnosis of asthma).^32^

Uncertainties in differentiating between asthma and COPD are reflected by the fact that there are no universally accepted criteria for the diagnosis of asthma-COPD overlap syndrome. A limitation of our data in this respect is that FEV1 measurements after bronchodilatation were not possible, which may have contributed to the high overlap rate and the high prevalence of asthma in older individuals (50–60 years).

Generally, the present study found a higher prevalence of asthma defined by prior diagnosis and clinical symptoms compared to GBD data for Nicaragua, including women.^17^ However, the female asthma prevalence in the present study was comparable to the data of Clark *et al*. of 12.7% for wood smoke-exposed Nicaraguan women in the same region of Granada (*Table 5*), which may suggest an additional role of biomass exposure on asthma morbidity.^4^

The age-adjusted prevalence for chronic bronchitis was twice as high in older individuals (50–69 years) compared to younger adults, as expected. Obesity had nearly the same prevalence compared to the WHO data, but did not have a significant confounding effect on chronic bronchitis.^36^ The same is true regarding smoking status in contrast to developed countries.

In addition to the generally high exposure levels of all participants, the present study found a difference in the prevalence of chronic bronchitis between participants with and without elevated exposure (those mainly responsible for cooking in a household). Participants with elevated exposures showed a chronic bronchitis prevalence of 19.5% (n=32) versus 5.4% (n=2) in participants without elevated exposure. Due to the small sample size of the last group, this finding may be biased. However, participants with elevated exposure had a markedly higher prevalence for chronic bronchitis than the total study population. A study in Pakistan indicated that wood smoke has one of the highest associations with chronic bronchitis compared to other solid fuels. The authors found a significant association between wood smoke exposure due to serving as primary household cook and chronic bronchitis among exposed women compared to a control group using liquefied petroleum gas with an odds ratio of 2.51.^37^ A Bolivian cross-sectional study of 100 adults > 20 years analyzed the prevalence of chronic bronchitis, comparing indoor and outdoor cooking participants. Prevalence, as expected, was higher for the indoor cooking group, above the 13% prevalence for the outdoor cooking group.^38^

There was an increased prevalence of chronic bronchitis in inhabitants of Region II compared to Region I. Possible reasons include higher ambient air pollution and lower socioeconomic status in these neighbourhoods as indicated by national data: the population rate living in extreme poverty was 16.8% in Region I and 26.1% in Region II.^22^–^24^ Poverty is known to be linked with COPD.^39^–^42^ Gonzalez-Garcia *et al*. reported a higher prevalence of chronic bronchitis in Colombia in individuals with low levels of education (8.2 vs. 4.6%).^19^ Furthermore, the rate of wood use for cooking was 25% in Region I, but 33%-64% in Region II in Nicaragua.^22^–^24^ The 2012 report of the comparative risk assessment from the Global Burden of Disease Study 2010 estimated that HAP contributes 16% of ambient air pollution.^5^ There is no official data on PM in Nicaragua, but in Colombia there was a higher prevalence of chronic bronchitis in two cities with higher levels of annual mean PM_2.5_ compared to the other three (smaller) cities with lower PM levels.^26^

This study has several limitations, including limited sample size, cross-sectional design and selection bias, as it was possible that more symptomatic individuals were motivated to participate. We were not able to consider long-time exposure to wood smoke which would better evaluate lifetime exposure. Direct measurement of ambient air and household pollutants would also contribute to a better understanding of this issue. Strengths of the present study were the combination of data on clinical symptoms, respiratory scores and lung function in a country where data on respiratory health is scarce.

## Conclusions

In the present study, a high prevalence of chronic bronchitis and COPD was found in a relatively young, wood smoke-exposed population compared to data from the general population in Nicaragua. Future studies should implement an overall exposure index and include more detailed data on socioeconomic status and environmental hazards to better understand the impact of HAP on respiratory health.
