# Supplementary material for: Prevalence of Chronic Bronchitis and Respiratory Health Profile of a Population Exposed to Wood Smoke in Nicaragua
Source: J Health Pollut. 2020 May 26;10(26):200607. doi: 10.5696/2156-9614-10.26.200607 (PMC7269325; doi:10.5696/2156-9614-10.26.200607)
Supplement: Supplementary file 1 [file Maas_Supplemental.doc]

**Supplemental Material**

**Baseline Questionnaire of Pulmonary Study in Nicaragua**

ENGLISH VERSION

**A. INTRODUCTION AND CONSENT:**

| **Question** | | **Answer** | **Code** |
| --- | --- | --- | --- |
| A1 | Name of village |  |  |
| A2 | Ethnic group |  |  |
| A3 | ID (surname,  given names) |  |  |
| A4 | Date of birth |  |  |
| A5 | Interviewer ID |  |  |
| A6 | Date | dd/mm/yy |  |
| A7 | Consent | No = 1  Yes = 2 |  |

**GENERAL MEDICAL INFORMATION:**

| **Question** | | **Answer** | **Code** |
| --- | --- | --- | --- |
| A10 | Weight | In kg |  |
| A11 | Height | In m |  |
| A12 | Profession |  |  |
| A13 | Do you have allergies? | No = 1  Yes = 2 |  |
| A14 | If Yes: which allergies do you have? | Name allergies |  |
| A15 | If Yes: Do you have allergies related to your profession? | Name allergies |  |

**CHRONIC RESPIRATORY SYMPTOMS**

**B. COUGH:**

| **Question** | | **Answer** | **Code** |
| --- | --- | --- | --- |
| B1 | Do you cough or have you coughed a lot?  **If “NO”, go to section C (Phlegm)** | No = 1  Yes = 2 |  |
| B2 | Do you cough or have you coughed when getting up in the morning? | No = 1  Yes = 2 |  |
| B3 | **If Yes:** How long have you been coughing when getting up in the morning? | Less than 3 months = 1  Around 3 months = 2  More than three months  = 3 |  |
| B4 | **If Yes:** During this time, how often do you cough when getting up in the morning? | Frequently = 1  Once in a while = 2 |  |
| B5 | Do you cough or have you coughed during the day? | No = 1  Yes = 2 |  |
| B6 | **If Yes:** How long have you been coughing during the day? | Less than 3 months = 1  Around 3 months = 2  More than three months  = 3 |  |
| B7 | **If Yes:** During this time, how often do you cough during the day? | Frequently = 1  Once in a while = 2 |  |
| B8 | Do you cough or have you coughed during the night? | No = 1  Yes = 2 |  |
| B9 | **If Yes:** Since how long ago have you been coughing during the night? | Less than 3 months = 1  Around 3 months = 2  More than three months  = 3 |  |
| B10 | **If Yes:** During this time, how often do you cough during the night? | Frequently = 1  Once in a while = 2 |  |
| B11 | Have you coughed up blood? | No = 1  Yes = 2 |  |

**C. Phlegm:**

| **Question** | | **Answer** | **Code** |
| --- | --- | --- | --- |
| C1 | Do you produce or have you produced a lot of phlegm?  **If “NO” go to section D (Periods of Cough with Phlegm)** | No = 1  Yes = 2 |  |
| C2 | Do you produce or have you produced phlegm when getting up in the morning? | No = 1  Yes = 2 |  |
| C3 | **If, Yes:** For how long have you been producing phlegm when getting up in the morning? | Less than 3 months = 1  Around 3 months = 2  More than three months  = 3 |  |
| C4 | **If, Yes:** During this time, how often do you produce phlegm when getting up in the morning? | Frequently = 1  Once in a while = 2 |  |
| C5 | Do you produce or have you produced phlegm during the day? | No = 1  Yes = 2 |  |
| C6 | **If, Yes:** How long ago have you been producing phlegm during the day? | Less than 3 months = 1  Around 3 months = 2  More than three months  = 3 |  |
| C7 | **If, Yes:** During this time, how often do you produce phlegm during the day? | Frequently = 1  Once in a while = 2 |  |
| C8 | Do you produce or have you produced phlegm during the night? | No = 1  Yes = 2 |  |
| C9 | **If, Yes:** How long ago have you been producing phlegm during the night? | Less than 3 months = 1  Around 3 months = 2  More than three months  = 3 |  |
| C10 | **If, Yes:** During this time, how often do you produce phlegm during the night? | Frequently = 1  Once in a while = 2 |  |
| C11 | **Have you had chronic cough with mucus production for at least three months of two or more consecutive years?** | No= 1  Yes = 2 |  |

**If the answer is “No” for questions B.1 and C.1: GO TO Section E (Asthma, Rhinitis and Eczema).**

**If the answer is “Yes” for questions B.1 and/or C.1: CONTINUE with Section D (Periods in Which Cough and Phlegm Get Worse).**

D. **PERIODS IN WHICH COUGH AND PHLEGM GET WORSE:**

| **Question** | | **Answer** | **Code** |
| --- | --- | --- | --- |
| D1 | During the past 12 months, have you had periods in which cough and phlegm **GET WORSE**?  **If NO, go to section E (Asthma, Rhinitis and Eczema)** | No = 1  Yes = 2 |  |
| D2 | **If, Yes:** How long has it been since you had periods in which cough and phlegm **GET WORSE***?* | A few days =1  One to two weeks =2  Three weeks or more  =3 |  |
| D3 | **If, Yes:** During the last 12 months, have you had more than one of these periods in which cough and phlegm **GET WORSE**? |  |  |
| D4 | Have you had difficulty breathing during the periods in which the cough or phlegm **GET WORSE**? | No = 1  Yes = 2 |  |

**E. ASTHMA, RHINITIS AND ECZEMA:**

| **Question** | | **Answer** | **Code** |
| --- | --- | --- | --- |
| E1 | Have you experienced a whistling sound in your chest with breathing?  **_If the answer is NO, go to E3** | No = 1  Yes = 2 |  |
| E2 | During the past 12 months, have you experienced periods in which the whistling sound in your chest with breathing got worse? | No = 1  Yes = 2 |  |
| E3 | During the past 12 months, have you ever woken up in the morning with the sensation of a pressure on your chest? | No = 1  Yes = 2 |  |
| E4 | Has the doctor or nurse ever diagnosed you with asthma? | No = 1  Yes = 2 |  |
| E5 | Have you ever had an itchy rash on your skin that appeared and disappeared for periods that lasted a total of at least 6 months?  **_If the answer is “NO”, go to question E8** | No = 1  Yes = 2 |  |
| E6 | Have you had this itchy rash at any moment during the last 12 months? | No = 1  Yes = 2 |  |
| E7 | Have you ever had this itchy rash in any of the following places? **(a)** Fold of your elbow, **(b)** Behind your knee, **(c)** Fold of your ankle, **(d)** Below your buttocks, or **(e)** Around your neck, ears and eyes  **(a)(b)(c)(d)**  **(e)** | No = 1  Yes = 2 |  |
| E8 | Have you ever had problems with sneezing, mucus or blocked nose when you **DID NOT** have a cold or flu?  **_If the answer is “NO”, go to question F1** | No = 1  Yes = 2 |  |
| E9 | During the past 12 months, have you had problems with sneezing, mucus or blocked nose when you **DID NOT** have a cold or flu? | No = 1  Yes = 2 |  |

**F. HEADACHE AND BURNING EYES:**

| **Question** | | **Answer** | **Code** |
| --- | --- | --- | --- |
| F1 | During the past month, have you had headaches? | No = 1  Yes = 2 |  |
| F2 | **If, Yes:** How often have you had headaches during this time? | Every day = 1  Most days of the week =2  A few days per week = 3  Once per week = 4  Less than once per week = 5 |  |
| F3 | **If, Yes:** How strong are the headaches? | Very strong = 0  Average = 1  Mild = 2 |  |
| F4 | During the past month, have you had burning eyes,  watery eyes**?** | No = 1  Yes = 2 |  |
| F5 | **If, Yes:** During this time, how often have you had burning eyes or watery eyes? | Every day = 1  Most days of the week =2  A few days per week = 3  Once per week = 4  Less than once per week = 5 |  |
| F6 | **Ask and observe:**  If your eyes water, what is the secretion like? | Clear, aqueous = 1  Yellow, green, sticky = 2 |  |
| F7 | **If the answer = 2:** the secretion is yellow or greenish and sticky, does it make it so you cannot open your eyes when you wake up?  ***If, Yes =2:***  **If, Yes*: Refer to Health Center*** | No = 1  Yes = 2 |  |

**G. BACK PAIN:**

| **Question** | | **Answer** | **Code** |
| --- | --- | --- | --- |
| G1 | Have you had back pain during the past month?  **If the answer is “No”, go to section H** | No = 1  Yes = 2 |  |
| G2 | **If, Yes:** During this time, how often have you had back pain? | Every day = 1  Most days of the week =2  A few days per week = 3  Once per week = 4  Less than once per week = 5 |  |
| G3 | **If, Yes:** What things make your back hurt more? | Carrying Wood = 1  Washing Clothes = 2  Cooking = 3  Other (specify) = 4 |  |
| G4 | **If, Yes:** Has the back pain been so strong that that you have stopped doing your activities? | No = 1  Yes = 2 (specify in the following box) |  |
| G5 | Describe how the pain affects your activities: | |  |

**H. TRAFFIC/ TOBACCO SMOKING:**

| **Question** | | **Answer** | **Code** |
| --- | --- | --- | --- |
| **H1** | From Monday to Friday, how frequently do trucks pass by the roads where you live? | Never = 1  Almost never =2  A few times during the day = 3  Almost all day = 4 |  |
| **H2** | Do you smoke cigarettes or cigars? | No = 1  Yes = 2 |  |
| **H3** | **If, Yes**: *How many cigarettes per day do you usually smoke?* | # Cigarettes/day |  |
| **H4** | Is there someone else who smokes  **inside** the house and/or the kitchen? | No = 1  Yes = 2 |  |

**J. Additional Questions/ Clinical Signs of Neoplasia**

| **Question** | | **Respuesta** | **Código** |
| --- | --- | --- | --- |
| J 1 | Have you ever been diagnosed with a chronic disease by a physician or nurse? | No = 1  Yes = 2 |  |
| J 2 | **If yes: which disease?** | Write the diseases |  |
| J 3 | Are you the person that normally cooks at home? | No = 1  Yes = 2  Sometimes = 3 |  |
| J 4 | How much time do you spend near the fire per day? | 1 hour = 1  2-3 hours = 2  4-6 hours =3  over 6 hours = 4 |  |
| J 5 | Do you use mosquito coil inside the house and/or at night or do you burn car tires to fight mosquitos? | No = 1  Yes = 2 |  |
| J 6 | Have you head fever in the last couple of weeks (over 38°C)? | No = 1  Yes = 2 |  |
| J 7 | Have you had unexplained weight loss in the last months (over 10%)? | No = 1  Yes = 2 |  |
| J 8 | **If yes**: How many kilos did you lose? | in kg |  |
| J 9 | Have you head night sweats lately? (more than normal) | No = 1  Yes = 2 |  |

**COPD Assessment Test (CAT)**

In the following questions please choose a point between 0 and 5: as an example: 0 corresponds to “I never cough”, 1 corresponds to “I sometimes cough”, 2 corresponds to “I cough a little”, 3 corresponds to “I often cough”, 4 corresponds to “I cough very often” and 5 corresponds to “I cough all the time” (Mark with a cross):

| CAT1 | **I never cough** | [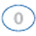](javascript:clicked(1,0);) [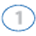](javascript:clicked(1,1);) [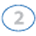](javascript:clicked(1,2);) [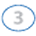](javascript:clicked(1,3);) [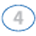](javascript:clicked(1,4);) [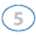](javascript:clicked(1,5);) | **I cough all the time** |
| --- | --- | --- | --- |
| CAT2 | **I have no phlegm (mucus) in my chest at all** | [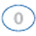](javascript:clicked(1,0);) [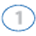](javascript:clicked(1,1);) [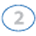](javascript:clicked(1,2);) [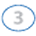](javascript:clicked(1,3);) [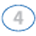](javascript:clicked(1,4);) [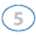](javascript:clicked(1,5);) | **My chest is full of phlegm (mucous)** |
| CAT3 | **My chest does not feel tight at all** | [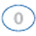](javascript:clicked(1,0);) [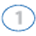](javascript:clicked(1,1);) [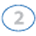](javascript:clicked(1,2);) [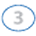](javascript:clicked(1,3);) [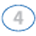](javascript:clicked(1,4);) [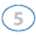](javascript:clicked(1,5);) | **My chest feels very tight** |
| CAT4 | **When I walk up a hill or a flight of stairs I am not out of breath** | [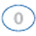](javascript:clicked(1,0);) [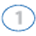](javascript:clicked(1,1);) [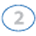](javascript:clicked(1,2);) [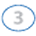](javascript:clicked(1,3);) [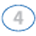](javascript:clicked(1,4);) [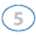](javascript:clicked(1,5);) | **When I walk up a hill or a flight of stairs I am completely out of breath** |
| CAT5 | **I am not limited in doing any activities at home** | [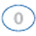](javascript:clicked(1,0);) [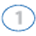](javascript:clicked(1,1);) [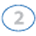](javascript:clicked(1,2);) [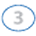](javascript:clicked(1,3);) [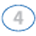](javascript:clicked(1,4);) [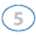](javascript:clicked(1,5);) | **I am completely limited in all activities at home** |
| CAT6 | **I am confident leaving my home despite my lung condition** | [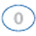](javascript:clicked(1,0);) [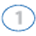](javascript:clicked(1,1);) [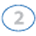](javascript:clicked(1,2);) [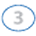](javascript:clicked(1,3);) [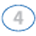](javascript:clicked(1,4);) [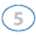](javascript:clicked(1,5);) | **I am not confident leaving my home at all because of my lung condition** |
| CAT7 | **I sleep soundly** | [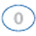](javascript:clicked(1,0);) [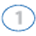](javascript:clicked(1,1);) [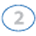](javascript:clicked(1,2);) [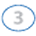](javascript:clicked(1,3);) [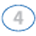](javascript:clicked(1,4);) [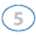](javascript:clicked(1,5);) | **I do not sleep soundly because of my lung condition** |
| CAT8 | **I have a lot of energy** | [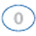](javascript:clicked(1,0);) [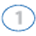](javascript:clicked(1,1);) [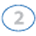](javascript:clicked(1,2);) [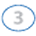](javascript:clicked(1,3);) [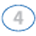](javascript:clicked(1,4);) [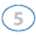](javascript:clicked(1,5);) | **I have no energy at all** |

**mMRC (Modified Medical Research Council) Dyspnea Scale**

If you have difficulties with breathing please choose the best response (only one) to describe your shortness of breath

| 0 | I only get breathless with strenuous exercise |
| --- | --- |
| 1 | I get short of breath when hurrying across a level path or walking up a slight hill |
| 2 | I walk slower than people of the same age across a level path because of breathlessness or have to  stop for breath when walking at my own pace on a level path |
| 3 | I stop for breath after walking about 100 yards or after a few minutes on a level path |
| 4 | I am too breathless to leave the house or I am breathless when dressing |

**THANK INTERVIEWEE FOR THEIR PARTICIPATION – END OF INTERVIEW**

**Interview**

Interviewer Initials:

Interviewer Signature:

**Interview Check**

Supervisor Signature:

Date of check:
